# Tracking tripartite interaction dynamics: isolation, integration, and influence of bacteriophages in the *Paraburkholderia-Dictyostelium discoideum* symbiosis system

**DOI:** 10.3389/fmicb.2025.1537073

**Published:** 2025-05-02

**Authors:** Susanne DiSalvo, Negar Maness, Andrew Braun, My Tran, Andrew Hofferkamp

**Affiliations:** Department of Biological Sciences, Southern Illinois University Edwardsville, Edwardsville, IL, United States

**Keywords:** bacteriophage, symbiosis, *Dictyostelium discoideum*, amoeba, *Paraburkholderia*

## Abstract

**Introduction:**

Bacteriophages influence interactions between bacterial symbionts and their hosts by exerting parasitic pressure on symbiont populations and facilitating bacterial evolution through selection, gene exchange, and prophage integration. Host organisms also modulate phage-bacteria interactions, with host-specific contexts potentially limiting or promoting phage access to bacterial symbionts or driving alternative phenotypic or evolutionary outcomes.

**Methods:**

To better elucidate tripartite phage-bacteria-host interactions in real-time, we expanded the *Dictyostelium discoideum-Paraburkholderia* symbiosis system to include *Paraburkholderia*-specific phages. We isolated six environmental *Paraburkholderia* phages from soil samples using a multi-host enrichment approach. We also identified a functional prophage from monocultures of one of the *Paraburkholderia* symbiont strains implemented in the enrichment approach. These phages were evaluated across all three amoeba-associated *Paraburkholderia* symbiont species. Finally, we treated *Paraburkholderia* infected amoeba lines with select phage isolates and assessed their effects on symbiont prevalence and host fitness.

**Results:**

The isolated phages exhibited diverse plaquing characteristics and virion morphologies, collectively targeting *Paraburkholderia* strains belonging to each of the amoeba-symbiotic species. Following amoeba treatment experiments, we observed that phage application in some cases reduced symbiont infection prevalence and alleviated host fitness impacts, while in others, no significant effects were noted. Notably, phages were able to persist within the symbiont-infected amoeba populations over multiple culture transfers, indicating potential long-term interactions.

**Discussion:**

These findings highlight the variability of phage-symbiont interactions within a host environment and underscore the complex nature of phage treatment outcomes. The observed variability lays the foundation for future studies exploring the long-term dynamics of tripartite systems, suggesting potential mechanisms that may shape differential phage treatment outcomes and presenting valuable avenues for future investigation.

## Introduction

While phages exhibit relative biological simplicity, their significance within microbial ecosystems is profound, both in terms of their abundance and functional importance (Clokie et al., 2011; Suttle, 2005). Not only do they play a pivotal role in shaping microbial assemblages, but they also serve as significant drivers of bacterial evolution (Hendrix, 2014; Koskella and Brockhurst, 2014). Efforts to comprehend phage diversity and function within ecosystems has expanded from aquatic (Suttle, 2007) to soil ecosystems (Gómez and Buckling, 2011; Srinivasiah et al., 2008), and finally, to host-associated (biotic) microbiomes (Avellaneda-Franco et al., 2023; Chatterjee and Duerkop, 2018; Duerkop et al., 2018; Koskella and Taylor, 2018; Marbouty et al., 2021; Wilde et al., 2024; Zuppi et al., 2022). The inherent complexity of some of these systems challenges our ability to directly interrogate the contribution of phages to the systems composition and function. However, evidence emphasizes that phages are powerful players that, even at the individual level, can direct large-scale changes in microbial communities. This recognition underpins the core concept of phage therapy, where phages are harnessed as treatments for bacterial infections. In a straight-forward scenario, phage administration selectively reduces the population of the bacterial pathogen, and any phage-resistant mutants can be targeted by co-evolved phages or the host immune system. Further, resistance trade-offs may be beneficial to treatment, such as reduced virulence or enhanced antibiotic susceptibility (Chan et al., 2016; Majkowska-Skrobek et al., 2021; Zhang et al., 2022). However, direct (i.e., phage-mediated genetic modifications) and indirect (i.e., expansion of opportunistic pathogens) detrimental trade-offs are also possible (Addy et al., 2012; Oechslin, 2018; Zhang et al., 2022). Although the selection of highly lytic phages helps mitigate this, context-dependent characteristics are also likely to be relevant to treatment outcomes, underscoring the need to understand the diversity of phage-bacteria interactions in eukaryotic host systems (Drulis-Kawa et al., 2012; Oromí-Bosch et al., 2023).

Phages are also relevant to the natural establishment and maintenance of beneficial relationships between eukaryotes and bacterial symbionts. For example, specific phage genotypes correspond to diverse traits associated with insect symbionts (Bordenstein and Bordenstein, 2016; LePage et al., 2017; Oliver et al., 2009; Pichon et al., 2012; Shropshire et al., 2018; Weldon et al., 2013), while phage enrichment in algae-associated bacterial communities may enhance host phenotypic plasticity by enabling rapid selective shifts in bacterial assemblages (Stiffler et al., 2024). Furthermore, in marine sponges, phages play a role in modulating the immune system, facilitating the uptake and stability of beneficial microbial partners (Horn et al., 2016; Jahn et al., 2019). Similar evidence of phage-associated immunomodulation has been demonstrated in many other systems (Dąbrowska et al., 2006; Duerkop et al., 2018; Łusiak-Szelachowska et al., 2020; Sweere et al., 2019) including germ-free models (Gogokhia et al., 2019), suggesting phages can influence eukaryotes via pathways not directly linked to bacterial intermediaries (Barr, 2019). Characteristics of eukaryote ecosystems may, in turn, structure phage-bacteria interactions. For example, anatomical, chemical, or cell-based features may restrict phage access to susceptible bacterial symbionts (Xu et al., 2016), retain phages for localized persistence (Almeida et al., 2019; Barr et al., 2013), or enhance phage clearance from host tissues (Marchi et al., 2023).

To broaden our toolkit for illuminating phage-bacteria-host dynamics, we sought to identify and integrate bacteriophages into the *Paraburkholderia-Dictyostelium discoideum* symbiosis system. The eukaryotic host in this system, *D. discoideum,* is a soil dwelling social amoeba that shares many traits with higher eukaryotes, making it a useful model organism for the study of cellular mechanisms (Annesley and Fisher, 2009; Bozzaro, 2013; Kessin, 2001; Martín-González et al., 2021) and host-pathogen interactions (Barisch et al., 2015; Bozzaro and Eichinger, 2011; Dunn et al., 2018; Steinert and Heuner, 2005; Thewes et al., 2019). During its single-cellular stage, vegetative *D. discoideum* amoeba phagocytose bacteria and multiply via binary fission. In amoeba-dense, food-scarce conditions, amoeba aggregate into a multicellular slug and migrate to a suitable location for fruiting body formation. Fruiting bodies consist of a long stalk of dead cells which support a globular sorus containing hardy spore cells which can be subsequently dispersed to new environments for germination. Throughout the social cycle, *D. discoideum* employs both cell-autonomous and innate immune-like defense mechanisms against potential pathogens (Chen et al., 2007; López-Jiménez et al., 2019; Zhang et al., 2016).

Despite these defenses, natural *D. discoideum* isolates are frequently found in association with bacterial symbionts (most notably *Chlamydiae* and *Paraburkholderia* species) which can establish intracellular infections in amoeba cells and persist through host development and dispersal cycles (Brock et al., 2011; DiSalvo et al., 2015; Haselkorn et al., 2021; Haselkorn et al., 2018). More broadly, members of the *Burkholderia*, *Paraburkholderia*, and *Caballeronia* genera encompass diverse species that engage in symbioses with a wide range of hosts, including plants, fungi, insects, and protists, where they can play roles ranging from mutualism to pathogenicity (Estrada-de Los Santos et al., 2018; Kaltenpoth and Flórez, 2020). Given their facultative nature and the tractability of their host amoeba, *Paraburkholderia* symbionts of *D. discoideum* are particularly amenable for study as they can be fluorescently labeled and co-cultured with amoeba to initiate and track infection processes and outcomes (Shu et al., 2018). Furthermore, symbionts isolated from natural isolates of *D. discoideum* belong to three species- *Paraburkholderia agricolaris, Paraburkholderia hayleyella,* and *Paraburkholderia bonniea* (listed in descending order of screening prevalence and strain diversity), representing an intriguing range of genotypic variation (Brock et al., 2018; DuBose et al., 2022; Haselkorn et al., 2018). These features enable symbiont phenotype comparisons which have emphasized connections between genotype and environmental context with infection patterns (i.e., symbiont abundance and intracellular penetrance) and symbiotic outcomes (i.e., mutualistic to pathogenic; Haselkorn et al., 2018; Khojandi et al., 2019; Miller et al., 2020).

Given the available assortment of *Paraburkholderia* symbiont strains, we reasoned that symbiont-specific phages could be isolated from the environment and employed to interrogate the interplay between phage and symbiont pairings within the context of the amoeba host ecosystem. Furthermore, the abundance and diversity represented by the *Paraburkholderia* genera broadly, which includes a striking array of environmental and symbiont species and genome characteristics, potentially promises a correlating richness in phages ripe for environmental extraction (Compant et al., 2008; Mullins and Mahenthiralingam, 2021). Indeed, a history of phage infection is evidenced by putative prophage sequences and potential phage defense mechanisms present in the majority of *Paraburkholderia* genomes assessed, and prophage induction has been successfully accomplished for at least one of these species (*Paraburkholderia terrae* strain BS437; Pratama et al., 2018; Pratama and van Elsas, 2017). Yet, reports describing the successful isolation of *Paraburkholderia* phages from environmental sources are limited, emphasizing a gap in connecting ecological and evolutionary signatures of *Paraburkholderia*-phage associations with their contemporary dynamics and consequences.

Here, we describe six environmental phage isolates specific to *Paraburkholderia* symbionts of *D. discoideum* (named Bonzo, Paranha, Balex, Scuba, Bagra, and Scugar), which were collectively isolated following a series of environmental screening attempts. Collectively, these phages were isolated from samples originating from Texas and South Carolina using a muti-bacterial host enrichment approach. As a consequence of the enrichment process (in which sample filtrates were inoculated with *Paraburkholderia* cultures to enable target phage amplification), we also identified a functional prophage (Prozo70) from one of our symbiont strains. Individual phage isolates exhibit distinct host-ranges and plaquing patterns on the tested *Paraburkholderia* strains, providing a spectrum of lysis phenotypes. Collectively, the environmental phage isolates are characteristic of members belonging to the Siphoviridae, Podoviridae, and Myoviridae families. From this starting collection, we selected a subset of isolates to investigate within the context of the *D. discoideum-Paraburkholderia* symbiosis system. We found that phage persistence and the consequence of phage treatment for host amoeba and their bacterial symbionts varies by culture context and across distinct phage isolate and symbiont strain pairings. These results demonstrate the potential promise contained within this simple system to track and elucidate outcome trajectories of tripartite interactions.

## Materials and methods

### Bacterial and amoeba culture conditions

Bacterial strains (Supplementary Table 1) were cultured on YG medium (0.5% Yeast Extract, 0.4% Glucose, 0.1% NaCl, with 1.5% agar for solid media) or, when grown with amoeba, on SM/5 agar medium [2 g glucose, 2 g Peptone, 0.2 g yeast extract, 0.49 g MgSO_4_, 1.9 g KH_2_P0_4_, 1.3 g K_2_HPO_4_-3H_2_0, 17 g agar (Formedium) per liter]. For plaquing assays, soft agar overlays were prepared by pouring 5 mL of melted YG top agar (0.6% agar) with 200 μL bacterial overnight cultures, over YG agar plates. Unless otherwise indicated, bacterial cultures were incubated at 24°C for all assays and liquid cultures were agitated at 150 rpm. To prepare bacterial suspensions for amoeba culturing, bacterial colonies were resuspended in KK2 buffer (2.25 g KH_2_PO_4_ and 0.67 g K_2_HPO_4_ per liter) and set to a final concentration of 1.5 OD_600_ nm.

To establish *Paraburkholderia* infections in amoeba cultures, 10^5^ *Dictyostelium discoideum* spores (strains QS18 or V12) were plated on SM/5 agar plates with 200 μL of a bacterial mixture containing 95% *Klebsiella pneumoniae* and 5% *Paraburkholderia* suspension by volume. To maintain uninfected or previously infected amoeba cultures, spores were plated with pure *K. pneumoniae* suspensions. Cultures were incubated face-up under low lights at 24°C with 50–70% humidity for 5–8 d to allow fruiting body development.

### Phage filtrate preparation from soil and culture samples

Surface soil (50–100 g, <8 inches deep) was collected from wooded sites in the U.S., including Edwardsville, IL; Atlanta, GA; St. Louis, MO; Houston, TX; and Bluffton, SC. Samples were suspended in YG broth (1 mL/ gram of soil) for 1 h at 100 rpm, centrifuged at 10 K rpm for 5 min, and filtered through a 0.22 μm filter. Five ml aliquots were removed and stored at 4°C as “pre-enriched” filtrates. The remaining filtrate was amended with 10x YG broth, inoculated with 100 μL of overnight liquid cultures from each of the indicated enrichment strains and incubated at 24°C for 24–48 h at 100 rpm. Samples enriched filtrates were prepared and stored as described above.

### Phage isolation

Sample filtrates were spotted on bacterial soft agar overlay plates and incubated for 24–48 h. Lawn clearance indicative of bacteriophage lysis was recorded and qualitatively assessed (0 = no lysis; 5 = transparent lysis zone). Lysis producing filtrates of interest were serially diluted in phage buffer (20 mM Tris–HCl pH 7.4, 100 mM NaCl, 10 mM MgSO₄) and plated on susceptible strains using the double agar layer method by either spotting 10 μL of filtrate dilutions on bacterial lawns or mixing 100 μL of filtrate with 200 μL of bacterial overnight culture and 5 mL YG top agar before pouring over YG agar plates. After 24–48 h of incubation, individual plaques were extracted using pipette tips, suspended in phage buffer, and re-plated on host lawns. This purification was repeated three times to obtain pure phage isolates.

### Phage amplification and enumeration

Phage stock serial dilutions were plated via the double agar layer method and incubated at 24°C for 24–48 h. Plates showing confluent lysis were incubated at room temperature for 1 h at 100 rpm with 3 mL of phage buffer added to suspend phage particles. The phage buffer from each plate was pooled into a Falcon tube, centrifuged at 10 K rpm for 5 min, and filtered through a 0.22 μm filter. Phage stocks were stored at 4°C and periodically sampled, serially diluted, and spotted on host lawns to quantify PFU titers.

### Host range characterization and efficiency of plating

Purified phage lysates were spot tested on bacterial soft agar overlays and plaque presence and morphology was assessed following incubation at 24°C for 24–48 h. To determine efficiency of plating (EOP), identical phage dilutions were spotted on bacterial lawns of each tested strain, and phage titers for each were quantified following incubation and plaque formation. The efficiency of plating was calculated by dividing the phage titer produced on the test strain by the phage titer produced on the reference strain and expressed as a percentage. Reference strains were selected based on their practical suitability as hosts for each tested phage (i.e., strains that effectively amplify the phage isolate).

### Phylogeny construction

ATP synthase beta chain (atpD) gene sequences corresponding to individual *Paraburkholderia* strains (for each of the *D. discoideum* symbiont strains), or to a species-representative strain (for some of the *Paraburkholderia* strains that do not form a symbiosis with *D. discoideum* when strain-specific sequences were unavailable) were imported into Mega11 from NCBI (see Supplementary Table 1 for GenBank accession numbers), aligned with MUSCLE, and used to construct a neighbor end-joining tree with 1,000 bootstrap replicates.

### Transmission electron microscopy

Processing and imaging of purified phage samples by transmission electron microscopy was performed by Wandy Beatty at the Washington University Molecular Microbiology Imaging Facility. Samples were fixed with 1% glutaraldehyde (Ted Pella Inc., Redding CA) and allowed to absorb onto freshly glow discharged formvar/carbon-coated copper grids for 10 min. Grids were then washed in dH_2_O and stained with 1% aqueous uranyl acetate (Ted Pella Inc.) for 1 min. Excess liquid was gently wicked off and grids were allowed to air dry. Samples were viewed on a JEOL 1200EX transmission electron microscope (JEOL United States, Peabody, MA) equipped with an AMT 8-megapixel digital camera (Advanced Microscopy Techniques, Woburn, MA).

### Phage stability and treatment assays

Phage stability on SM/5 plates was assessed by spreading 100 μL phage filtrate (10^8^ PFU/ml for a total of 10^7^ PFU’s) on the agar surface in duplicate. Samples were harvested for quantification after 24 h and 7 days. For quantifying phages on uninfected and *Paraburkholderia*-infected amoeba cultures, 100 μL phage filtrate (10^8^ PFU/ml), 100 μL spore suspension (10^6^ spores/ml, for a total of 10^5^ spores) from uninfected or infected amoeba lines, and 200 μL *K. pneumoniae* suspension (as previously described) were plated on SM/5 in duplicate (from identical sample pools, providing paired samples for two time-point assessments). One plate was harvested after 24 h, and the other was incubated for 1 week to allow fruiting body formation. 10^5^ spores were transferred from these developed plates to new SM/5 plates with *K. pneumoniae*, and incubated for an additional 24 h. Following incubation, sample filtrates were prepared by adding 4 mL of phage buffer with 0.1% Igepal to the agar surface to resuspend total plate contents. A 2 mL aliquot was collected, centrifuged at 10 K rpm for 2 min, filtered (0.2 μm), serially diluted, and spotted on host lawns (corresponding to infection condition strain) for PFU enumeration.

To assess phage treatment outcomes, spore suspensions were mixed with phage filtrates (or with 100 μL phage buffer for untreated controls) and plated (in duplicate) as described above. Following 1 week incubation, total plate contents from one plate were harvested for phage enumeration and the other plate was used to harvest spores for infection prevalence quantification and (for assessment of outcomes following several social cycle passages) to initiate new amoeba cultures (as described).

Infection prevalence was quantified from suspended spores using a BD-C6 Flow Cytometer. After gating spores, the percent of *Paraburkholderia* RFP-positive spores were determined from PE-A intensity histograms, with uninfected samples used to establish fluorescent and non-fluorescent spore boundaries. An Olympus Fluoview FV1000 confocal microscope was used to acquire representative images of spores (stained with 1% calcofluor and covered with a 2% agarose overlay on a glass-bottom dish) from the treatment conditions and imaged using the Plan Ap Oil 1.4NA 60x objective. The DAPI and Cy3 channels were used to image Calcofluor and RFP, respectively, with several Z-sections (step size of 0.5 μm) acquired at a 1,024 × 1,024 resolution taken for each sample and images were processed in FIJI (imagej.net/Fiji). Total PFU’s were quantified from plate suspensions as described previously, with pre-filtered suspension aliquots being reserved for spore quantification (via hemocytometer counts).

### Statistics

All statistical analyses were performed using R version 4.2.3. When the assumption of normality was met, comparisons between infection outcomes (e.g., total PFUs, spore counts, or RFP values), with infection and/or treatment condition as independent variables, were assessed using ANOVA. If normality was violated, the Kruskal-Wallis test was applied instead. Post-hoc comparisons among strains were conducted using Tukey’s test for significant ANOVA results or Dunn’s test for significant Kruskal-Wallis results. For pairwise comparisons in phage treatment transfer experiments (e.g., PFU and spore counts between two transfers within treatment conditions), the Wilcoxon rank-sum test was used.

## Results

### Phage isolation and phenotypic characterization

**Figure 1:**
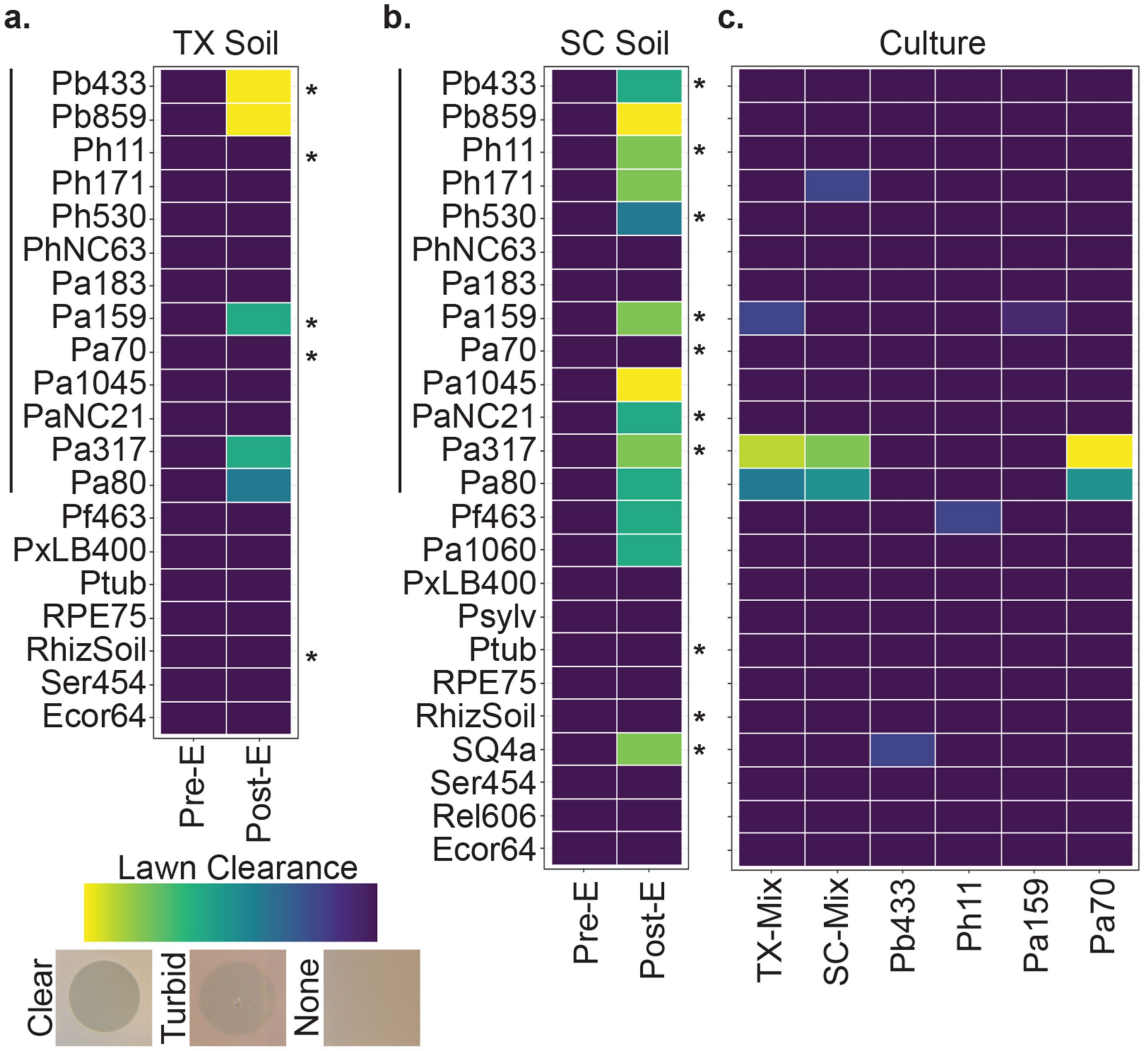
Phage screening. Tile plots representing lawn clearance from pre- and post-enriched filtrates generated from **(a)** Texas (TX) and **(b)** South Carolina (SC) soil samples, and of **(c)** culture filtrates from mixed- and mono-cultures representative of the enrichment conditions. The Y axis represents bacterial strains tested against the filtrate types indicated on the X axis. Left-hand vertical lines correspond to symbiont strains of *D. discoideum*, right-hand asterisks indicate each strain used in the multi-host enrichment condition. Observation of lysis zones were qualitatively scaled from no change (dark purple) to a full clearance (light yellow) in lawn thickness. See Supplementary Table 1 for a full list of bacterial strains.

To isolate bacteriophages specific to *Paraburkholderia* symbionts of *Dictyostelium discoideum*, we periodically collected and screened environmental samples throughout the United States. To improve detection of low-abundance phages, we implemented a multi-host enrichment method by inoculating sample filtrates with bacterial cultures and incubating for 24–48 h before preparing filtrates. Aliquots from sample filtrates collected before (pre-E) and after (post-E) enrichment with mixed *Paraburkholderia* cultures were spot tested on bacterial lawns and monitored for lysis plaques. While pre-enriched filtrates rarely produced clearance zones, post-enriched filtrates produced from Houston Arboretum (Texas) and Sun City (South Carolina) soil yielded plaques on several *Paraburkholderia* strains (Figure 1). Occasionally, faint lysis zones were observed from enriched filtrates from soil, lake, and sewage collected from other sites, but we were unable to purify symbiont-specific phages of interest from these samples.

To determine whether lysis plaques could be a product of prophages harbored by strains used for phage enrichment (rather than phages from the environmental sample), we tested filtrates from mixed and mono-cultures corresponding to the bacterial strains used during the phage enrichment process (i.e., filtrates produced from bacterial cultures without added environmental sources). From these, lysis zones were observed on two strains of *P. agricolaris* and subsequent testing of pure culture filtrates suggested that a prophage from another *P. agricolaris* strain, Pa70, was the likely source of this lysis pattern (Figure 1c). This conclusion was supported by bioinformatic identification of an intact prophage sequence in Pa70 using the PHASTER web server^1^ and consistent isolation of a Pa317-targeting, replication-competent, phage from Pa70 filtrates.

**Figure 2:**
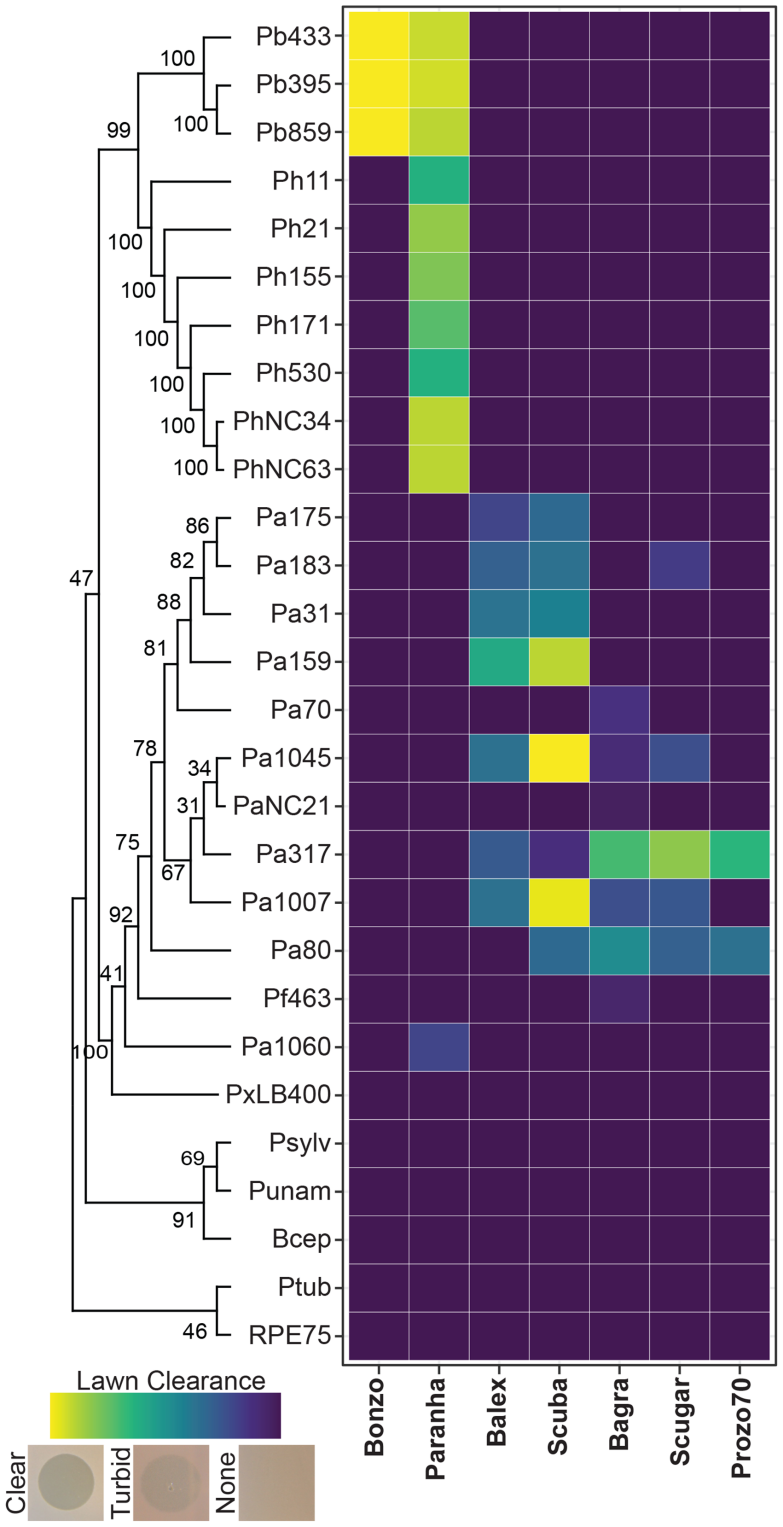
Host range and plaque clearance patterns. Tile plot representing lawn clearance values for the final phage isolates (x axis) when spot-tested on bacterial lawns (y axis). Test strains are arranged vertically according to phylogenetic relatedness based on aligned atpD gene sequences (Supplementary Table 1).

**Figure 3:**
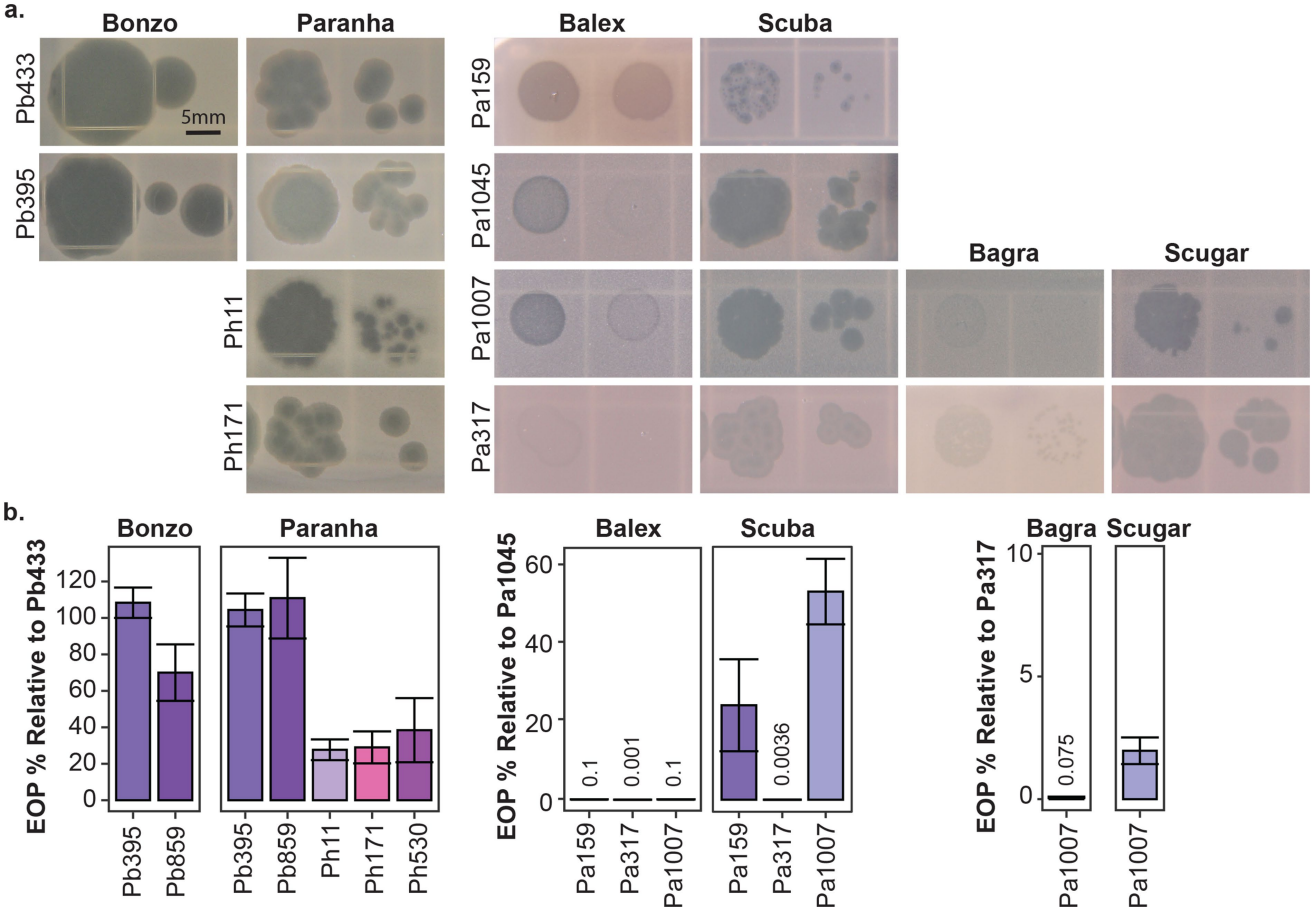
Plaque morphology and plating efficiency. Representative plaques **(a)** and efficiency of plating (EOP %) **(b)** for the environmental phage isolates on select bacterial host strains. Efficiency of plaquing is represented as the percentage of PFU/ml generated from test strains relative to PFU/ml generated from corresponding reference strains for each phage isolate (Pb433 for Bonzo and Paranha, Pa145 for Balex and Scuba, and Pa317 for Bagra and Scugar). Scale bar is representative for all images in **a**.

Seven *Paraburkholderia* symbiont targeting phage isolates (including the Pa70 prophage) were ultimately purified from these screening efforts (Figure 2). Most phages exhibited species-specific host ranges, except for Paranha, which infected strains of *P. bonniea* and *P. hayleyella* (Figures 2, 3). Bonzo only targeted *P. bonniea*, producing notably large, clear, plaques on host lawns. The other four environmental phages (Balex, Scuba, Bagra, and Scugar) and the Pa70 prophage (Prozo) targeted a subset of *P. agricolaris* strains, and, with the exception of clearer plaques produced by Scuba and Scugar on some hosts, plaques were predominantly turbid (Figures 2, 3). To assess the efficiency of plating across susceptible host bacteria, phage titers on bacterial test strains were compared to corresponding titers on a bacterial reference strain (chosen for each phage isolate based on phage amplification and plaque formation efficacy for each phage isolate). For Bonzo, amplification was least efficient on Pb859, while for Paranha, amplification was similarly efficient on all three *P. bonniea* hosts, which produced EOP values approximately five-fold higher than *P. hayleyella* host strains (Figure 3B). For *P. agricolaris* targeting phages, phage productivity was highest on Pa1045 for Balex and Scuba, and on Pa317 for Bagra and Scugar (Figure 3B).

**Figure 4:**
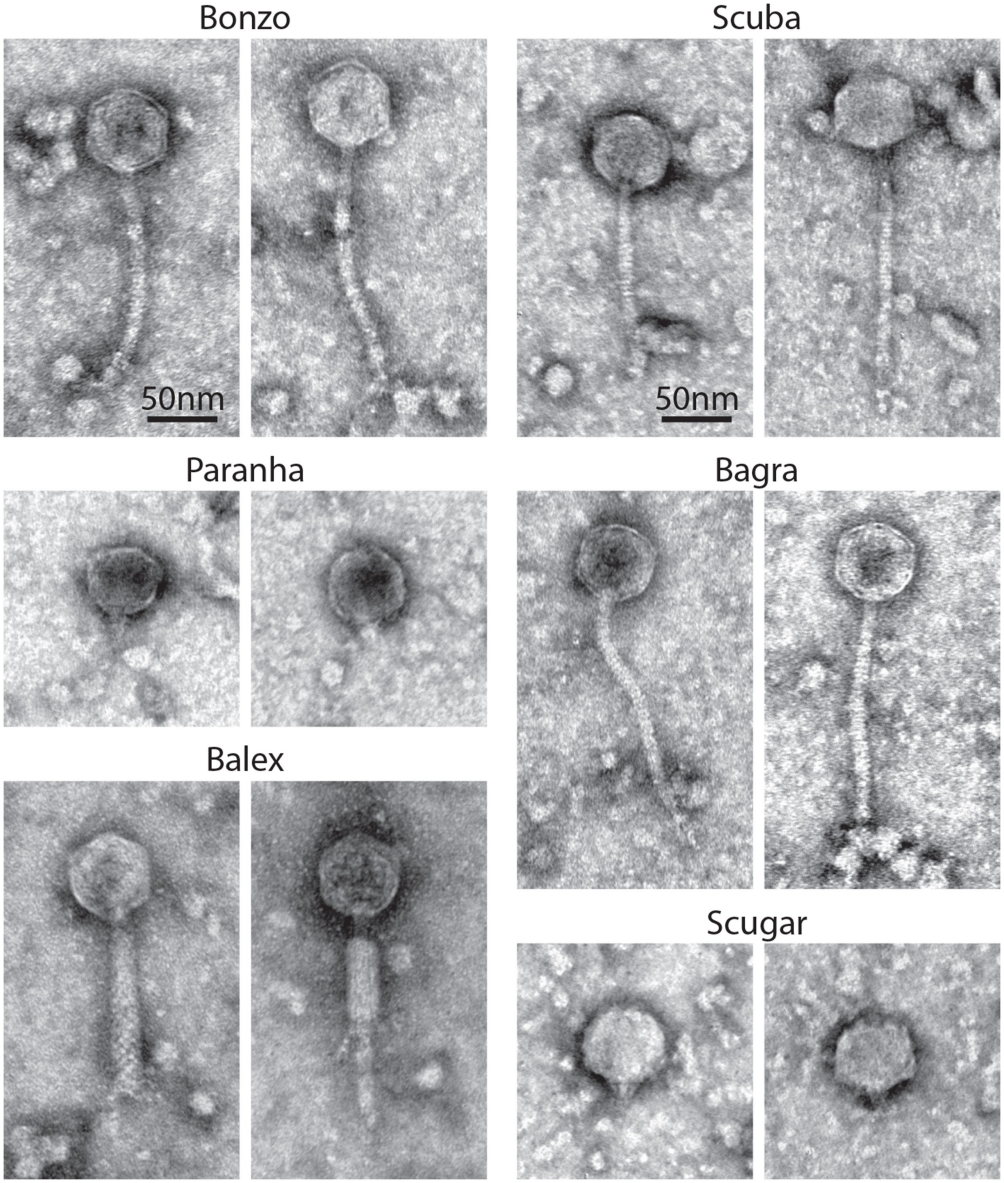
Phage virion morphology. Representative negatively stained transmission electron micrographs from high-titer filtrates of purified environmental phage isolates. Scale bars are representative for all images.

Transmission electron micrographs of high titer phage filtrates (>10^8^ PFU/ml) suggest that the environmental phages represent members of the Myoviridae, Siphoviridae, and Podoviridae (Figure 4). Contractile tails, characteristic of Myoviridae, (138.3 nm long on average) are readily visible on Balex, while long, flexible tails, characteristic of Siphoviridae, are visible on Bagra, Bonzo, and Scuba virions (148, 164, and 178 nm long on average, respectively). Paranha and Scugar virions were harder to identify, with Podoviridae resembling particles consisting of semi-rounded capsids with small tail-like protrusions being the only phage-like particles observed from multiple high-titer filtrate preparations. Average head capsid diameters ranged from 54 nm for Bagra to 63 nm Scugar, with all others between 56.3 and 57.3 nm.

### Phage stability in amoeba-associated culture conditions

**Figure 5:**
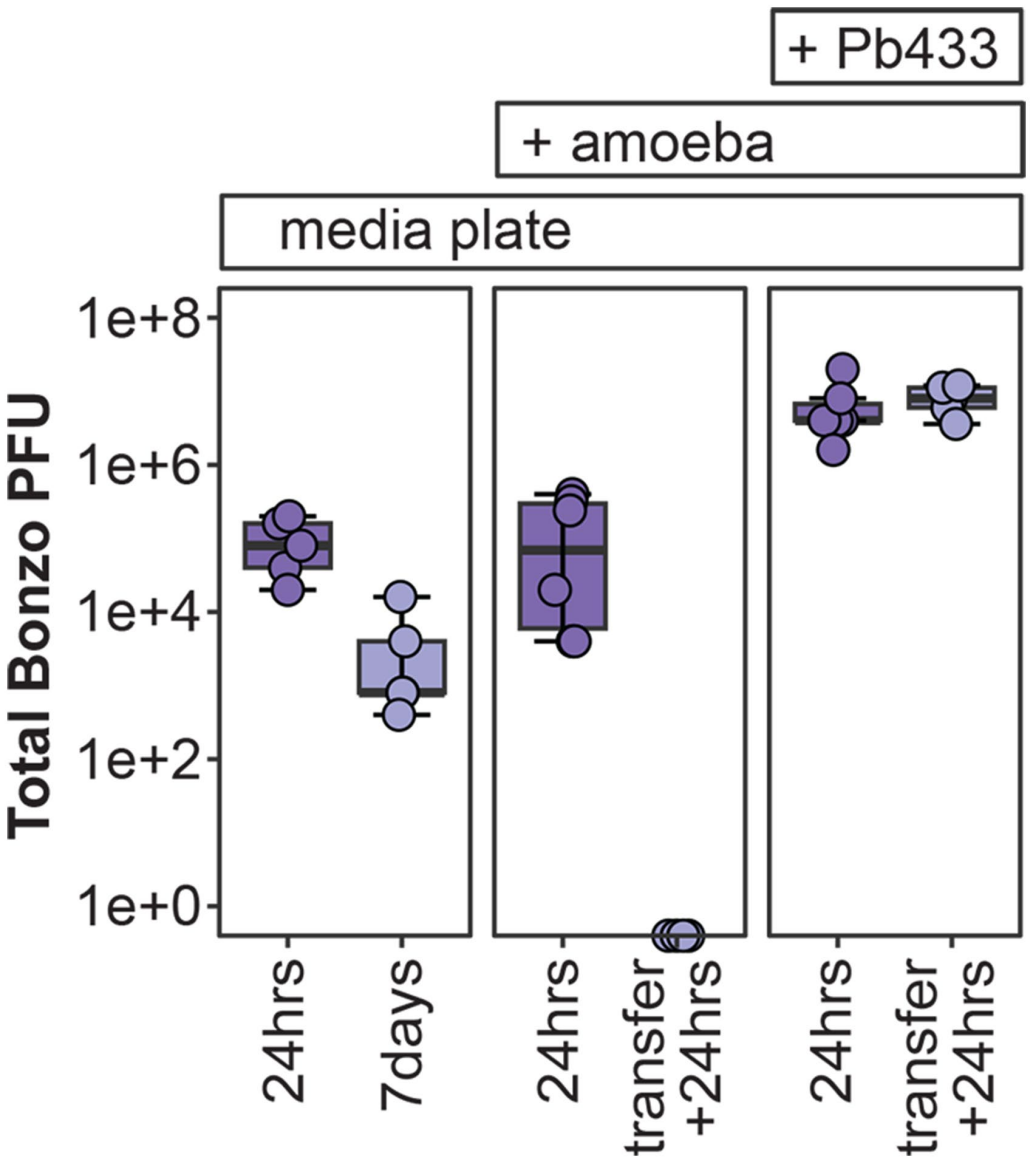
Bonzo stability in amoeba culture conditions. Total Bonzo PFU’s recovered following the indicated incubation periods on agar medium alone (media plate, left), or with *D. discoideum* amoeba that were either uninfected (+ amoeba, middle), or infected with Pb433 (+ Pb433, right). For amoeba co-culture conditions, transfer + 24 h represents re-culturing from *D. discoideum* spores developed from the initial plating conditions.

Next, we wanted to explore the impact of phage integration (i.e., phage “treatment”) in amoeba populations hosting phage susceptible *Paraburkholderia* symbionts (i.e., “infected” amoeba). However, before testing phage treatment outcomes for *Paraburkholderia* infected amoeba, we assessed our ability to detect and recover phages from plating contexts relevant to *D. discoideum* culturing conditions. To do this, we spread 10^7^ total Bonzo PFU’s on SM/5 plates alone, or with 10^5^ *D. discoideum* spores harvested from uninfected or Pb433 infected amoeba lines (and plated with *K. pneumoniae* as a food source). Plates were prepared in duplicate, and total PFU’s were quantified from filtrates prepared from plate contents harvested from two different time points per condition. Specifically, samples were collected 24 h after plating for all conditions, and 7 days after plating for SM/5-only samples, and, for uninfected and Pb433-infected amoeba culture conditions, after transferring spores developed from the duplicated samples (after 7 day incubations) to fresh SM/5 plates (with *K. pneumoniae*) and incubating for an additional 24 h (Figure 5).

Twenty-four hours after plating, total PFU’s recovered from all conditions were lower than the initial input of total PFU’s (10^7^), with SM/5-only, uninfected-amoeba, and Pb433-infected amoeba, plates yielding an average 10^5^, 1.65×10^5^_,_ and 6.93×10^6^ total PFU’s, respectively. Phage intercalation into the plating media likely limits the efficiency of recovery, accounting for some degree of phage loss following incubation. However, total PFU’s varied significantly across the conditions (df = 2, *F* = 5.55, *p* = 0.017); with Pb433-infected amoeba cultures yielding more PFU’s than either SM/5-only (*p* = 0.035) or uninfected-amoeba (*p* = 0.028) conditions (which did not significantly differ from one another, *p* = 0.99), suggesting that the presence of Pb433 bacterial symbionts carried with host spores improves/enables phage stability and/or amplification. To determine whether Bonzo could also persist throughout the amoeba social cycle and be carried to new environments during spore dispersal, we quantified total PFU’s from fresh cultures seeded with spores developed from the duplicated amoeba culture plates. Interestingly, we found that phages could only be recovered from the Pb433-infected amoeba lines, which yielded phage titers comparable to their pre-transfer counterparts. These results suggest that amoeba-associated phage co-dispersal is possible but requires facilitation by phage-susceptible symbionts.

### Phage, symbiont, and host outcomes in phage treated amoeba cultures

**Figure 6:**
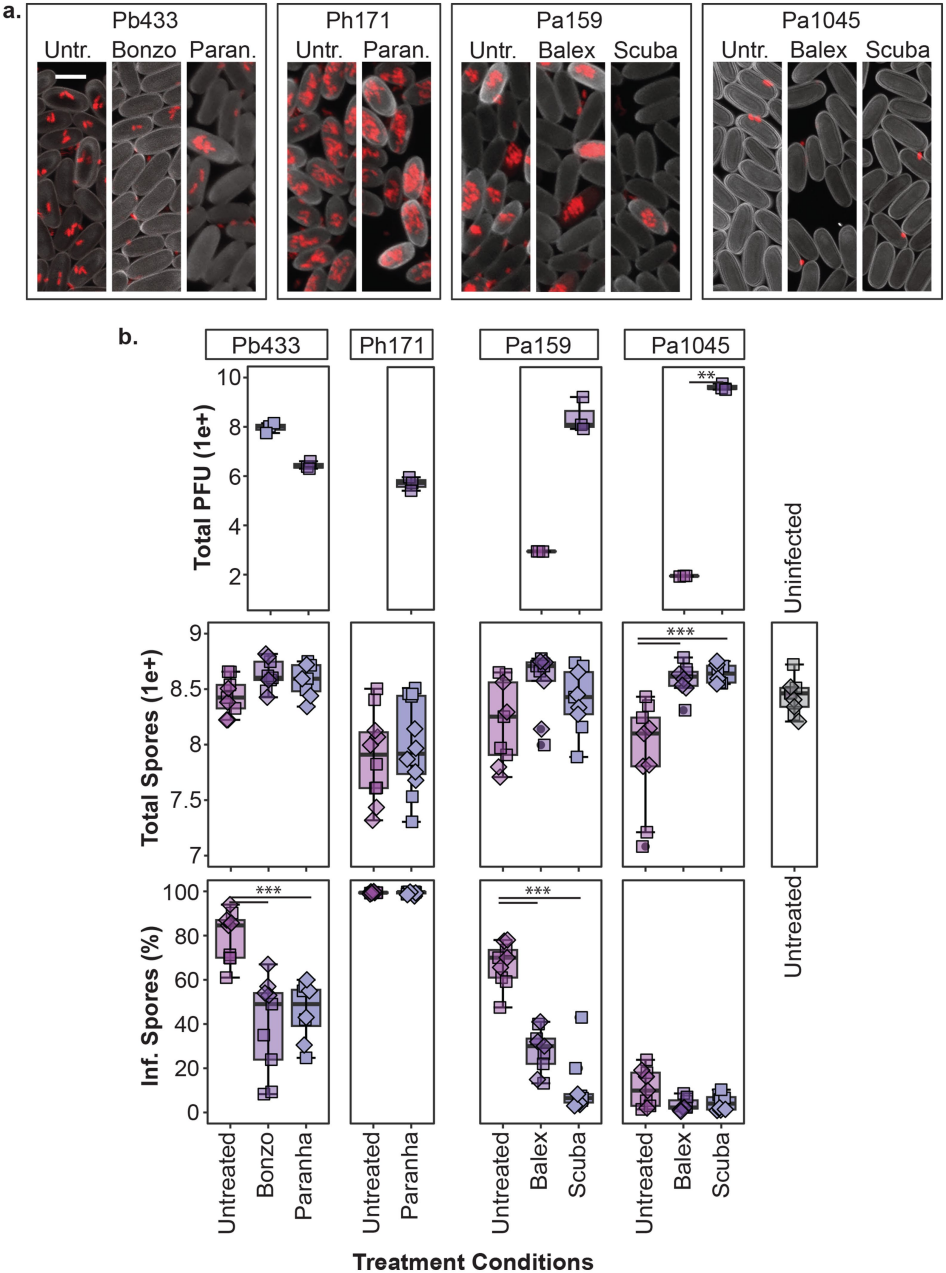
Phage treatment of *Paraburkholderia* infected amoeba. Representative confocal micrographs (z-projections) of sori contents **(a)** and box plots of total phage pfu’s, amoeba spores, and intracellular infection prevalence in spore populations (top, middle, and bottom panels, respectively) **(b)** from developed amoeba cultures (1 week incubation) for the indicated phage treatment and *Paraburkholderia* (rfp) infection combinations. Scale bar represents 5um. Plot points represent individual biological replicates from V12 (boxes) and QS18 (diamonds) amoeba lines (n ≥ 4 per strain). Asterisks represent statistically significant differences between indicated groups (**p-value *< 0.01,* ***p < 0.001).

To assess the impact of phages on host-symbiont dynamics, we established *Paraburkholderia* infected amoeba host lines and inoculated (i.e., treated) these lines with select phage isolates. Each amoeba host line was infected with one of the following four rfp-labeled *Paraburkholderia* symbiont strains: Pb433, Ph171, Pa1045, or Pa159. These host lines were subsequently treated with one of the following four phage isolates: Bonzo, Paranha, Balex, or Scuba, according to the susceptibility of the symbiont infection strain to the phage isolate as indicated by plaque formation on bacterial lawns (Figure 3). Treatment consisted of plating 10^5^ amoeba spores with 10^7^ phage PFU’s on SM/5 agar medium (supplemented with food bacteria) and incubating for 7 days to allow the completion of the amoeba social cycle and fruiting body formation. We set the phage dosage to reflect a treatment MOI of ~10, assuming that infected sori contents typically contain 10-fold *Paraburkholderia* cells than spore cells (estimating total bacterial symbiont cells plated to be ~10^6^). Although these numbers are likely to vary across infection conditions, preliminary treatment trails (Supplementary Figure 1) suggested that final outcomes do not vary noticeably according to starting phage dose in the tested condition (df = 3, *F* = 0.853, *p* = 0.5). After establishing treatment pairings and allowing the formation of fruiting bodies, we harvested plate contents to quantify total amoeba spores, *Paraburkholderia* symbiont infection prevalence in spore populations, and total phage PFU’s (Figure 6). We also visualized infection patterns in sori contents for representative samples using confocal microscopy (Figure 6A), which aligned with infection prevalence as quantified by flow cytometry (Figure 6B).

In line with previous reports, spore productivity is significantly impacted by *Paraburkolderia* infection status (df = 4, *F* = 5.175, *p* < 0.001), with Pa1045 and Ph171 (but not Pb433 or Pa159) resulting in a significant reduction in total spores compared to uninfected amoeba (*p* < 0.05; Figure 6B). While Paranha treatment did not significantly alleviate the fitness cost of Ph171 infections (df = 1, X^2^ = 0.43478, *p* = 0.509), phage treatment with either Balex or Scuba (*p* < 0.001) significantly improved total spore productivity for Pa1045 infected amoeba hosts (df = 2, *F* = 26.88, *p* < 0.0001). *Paraburkholderia* symbionts also establish different rates of intracellular infections in spore populations (df = 10, X^2^ = 90.057, *p* < 0.0001). As Pa1045 results in very low rates of intracellularly infected spores, capturing the potential significance of phage-mediated reductions in this parameter is limited. In contrast, Ph171 establishes high rates of intracellular infections, but Paranha treatment had no effect on this infection outcome. However, phage treatment with either Paranha or Bonzo phage isolates for Pb433, and with either Scuba or Balex for Pa159, significantly reduced the infection prevalence of these bacterial symbionts in host amoeba (*p* < 0.001). Finally, in regard to phage abundance in treated cultures, total pfu’s significantly varied across the collective treatments (df = p6, X^2^ = 19.35, *p* < 0.005), but within the same infection context, was only significantly different between Balex and Scuba in Pa1045 symbiont infected amoeba (*p* < 0.01).

**Figure 7:**
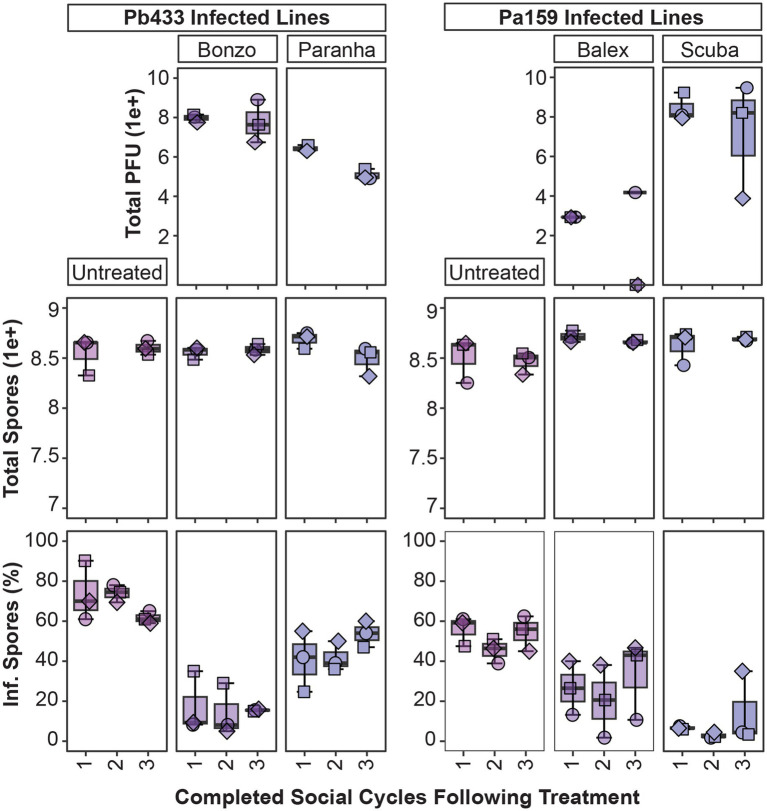
Tracking phage and symbiont infection parameters over multiple social cycles. Box plots of total phage PFU’s, amoeba spores, and intracellular infection prevalence from spore populations (top, middle, and bottom panels, respectively) after plating phage treated amoeba spores for the indicated infected amoeba lines, sampling after one social cycle (1), and serially transferring spores from harvested fruiting bodies from the prior cycle to complete a second (2), and third (3), round of development. For the second (2) cycle, data was collected for intracellular infection prevalence (bottom panel) but not for total PFU and spore production (top panels). Plot points represent individual biological replicates from QS18 amoeba lines (*n* = 3).

Next, we assessed symbiont infection dynamics over multiple amoeba social cycles following phage treatment for a subset of Pb433 and Pa159 infected amoeba host lines. To do this, we replated spores developed from phage treated amoeba lines on new media plates with a fresh supply of food bacteria. Following an additional round of incubation and fruiting body formation, we harvested developed sori, quantified intracellular symbiont infection prevalence, and transferred spores to new plates. After completion of this third social cycle (following phage addition) we again quantified symbiont infection prevalence, as well as total amoeba spores and phage PFU’s in developed amoeba host lines (Figure 7). Given that spore productivity is not significantly impacted by these symbionts, the lack of significant changes in this parameter following serial transfers was expected (df = 11, *F* = 1.8, *p* = 0.1). However, this also highlights that detrimental host fitness outcomes were not induced by phage treatment under these conditions. Furthermore, total Bonzo and Paranha PFU’s, and phage associated reductions in Pb433 symbiont infection prevalence in amoeba host lines, remained stable between the first and last social cycle [t(5) = 0.6, *p* = 0.56, and t(2) = 2.6, *p* = 0.121]. However, in the Pb433 infected amoeba host lines that were treated with Paranha, the infection prevalence of Pb433 in host spore populations appeared to be trending upwards with each transfer, corresponding with a slight downward trend in total Paranha PFU’s. For amoeba host lines infected with Pa159, symbiont infection prevalence rates for each phage treatment condition also remained relatively stable over the tested time-period (df = 5, X^2^ = 9.1205, *p*-value = 0.1044). However, Balex phage PFU’s could not be recovered from two out of three final amoeba host lines, and these exhibited higher Pa159 symbiont infection prevalence rates, suggesting phage abundance is inversely correlated with bacterial infection levels and that phage instability or loss may enable bacterial infections to rebound following phage treatment.

## Discussion

While our results demonstrate that *Paraburkholderia*-specific phages can be successfully isolated from soil, the effort we exerted toward this aim (i.e., reiterative screening attempts of water, soil, and sewage sampled collected from several states) was disproportional to our initial expectations. Presumably, the diversity and plasticity of *Paraburkholderia* species and their potential to occupy multiple overlapping habitats, should favor phage persistence, exchange, and diversification. Furthermore, at least some subset of prophage elements commonly associated with *Paraburkholderia* genomes should retain lytic potential, resulting in occasional environmental release (Pratama et al., 2018). While one study successfully induced a *Paraburkholderia* prophage, their attempts to isolate environmental phages failed to bear fruit, as has our search for additional sources describing the isolation of environmental *Paraburkholderia* phages (Pratama and van Elsas, 2017). However, accurately identifying relevant literature may be complicated by revolutions in phylogenomics and nomenclature, and the lag period toward consensus and broad application of new classifications. Specifically, the reclassification of the plant-beneficial-environmental clade of *Burkholderia* into a separate *Paraburkholderia* genus was proposed in 2014, and several new subdivision genera have since been proposed (Sawana et al., 2014; Dobritsa and Samadpour, 2016; Estrada-de Los Santos et al., 2018; Beukes et al., 2017). With this in mind, a prior study isolated an environmental phage active against a *Burkholderia* cinch-bug symbiont originally classified as a *Burkholderia* sp., but which is retrospectively likely to belong to the *Paraburkholderia* genera (Xu et al., 2016). Ultimately, it is unclear whether scarcity of *Paraburkholderia* phages in the literature is due to a limited number of attempted screens or limited success rates. Phage isolation is dependent on host species prevalence and characteristics, environmental context, and screening strategy (Hyman, 2019; Mattila et al., 2015; Nafarrate et al., 2020). In our own experience, local samples (~30-mile radius around Edwardsville, Illinois) have yet to yield phage isolates of interest, despite conducting over a dozen screens representing a range of seasons, sample-types, and sites. In contrast, our successful screens originated from soil collected from wooded locations in warmer climates (Texas and South Carolina), which were sent via mail for processing upon receipt, and kept aerated and moist prior to processing. Working from these successes, we hope to hone-in on sampling and screening conditions that prove most promising. However, if *Paraburkholderia* phage isolation is indeed more difficult than would be expected given the potential presence and distribution of putative bacterial hosts, this might point to an intriguing biological phenomenon inherent to *Paraburkholderia* bacteria (and/or their phages).

As a byproduct of our multi-host enrichment approach, we identified a functional prophage from the *P. agricolaris* Pa70 bacterial symbiont that could generate lysis plaques and amplify on the related Pa317 symbiont strain. This observation is intriguing and suggests several avenues for investigation regarding the potential functional role of this, and other putative prophages, on the genotypic and phenotypic diversity of *Paraburkholderia* strains, and how these contributions might relate to symbiotic vs. free-living traits*. P. agricolaris* appears to be the most prevalent and phenotypically diverse *Paraburkholderia* symbiont species of *D. discoideum* (DuBose et al., 2022), possessing a genome approximately twice the size (8.7 Mbp) of those of *P. bonniea* and *P. hayleyella* (4.1 Mbp; Brock et al., 2018; Noh et al., 2022). Horizontal gene transfer, including prophage integration-based transduction, has been posited as an important contributing factor for genome expansion and diversification (Arnold et al., 2022), and the presence of prophage sequences correlates to some extent with genome size (Casjens, 2003). However, this relationship is tenuous, as some of the largest bacterial genomes lack significant prophage signatures and are more likely the result of genome duplication (Casjens, 2003; Han et al., 2013). Nonetheless, the features and environmental prevalence of *P. agricolaris* symbionts suggests that they are more readily environmentally acquired and horizontally transmitted (as well as reseeded into the environment) by host amoeba (Shu et al., 2020; Shu et al., 2018). By extension, *P. agricolaris* strains may be more vulnerable to environmental phage exposure and as such more heavily shaped by phage-predation and modification than the other two symbiont species.

The phage collection isolated in this study allowed us to co-culture and track different phage isolate by bacterial symbiont pairings within amoeba host populations, highlighting the influence of individual members and alternative partner pairings in driving multi-partner interaction outcomes. A powerful feature of the system is that the natural variation in infection characteristics across symbiont strains enables investigations into how specific characteristics associated with bacterial symbionts and/or phage isolates interrelate to produce novel outcomes. The phage-susceptible bacterial symbionts in this study differed in their infection prevalence rates, intracellular infection density characteristics, and fitness costs for host amoeba. While Ph171 and Pb433 symbiont strains both produce high infection rates, host spores infected with Ph171 harbor more symbiont cells than host spores infected with Pb433, while total host spore productivity is reduced by Ph171 infections but unaffected by Pb433 infections. In contrast, symbiont Pa159 produces an average spore infection rate of 60%, compared to only 10.9% for Pa1045, and while they both tend to reduce spore production for host amoeba, this reduction was only significant for Pa1045. Because of some overlap in host-specificity, we could detect differences in outcomes between different phage isolates when targeting the same symbiont and between the same phage isolate when targeting different symbionts. Furthermore, given the potential for phage and symbionts to persist in amoeba cultures over multiple developmental cycles, initially subtle variations may exacerbate over time to reveal striking differences, as trends from our small-scale serial transfer experiment potentially portends (i.e., Paranha and Balex appeared to lose efficacy over time, in contrast to their Bonzo and Scuba counterparts).

In pairings where significant declines in infection prevalence were observed (Pb433, and Pa159), phage treatment did not completely eradicate intracellular infections in passaged hosts. For instance, Bonzo and Scuba phages were effective at reducing Pb433 and Pa159 infections, respectively, but low levels of infection persisted in the conditions tested. Additionally, for certain passaged amoeba host lines, phages appeared to have been lost entirely (e.g., Balex PFU’s were not recovered from two of three Pa159 amoeba host lines), or were on the verge of extinction (e.g., Scuba PFU’s declined in one of three amoeba lines). While immune mechanisms may improve phage therapy efficacy in mammals (Marchi et al., 2023), symbiont persistence in these phage treated amoeba lines does not rule out the possibility that primitive immune-like mechanisms in *D. discoideum* can act synergistically with phages. Indeed, as *Paraburkholderia* symbionts establish persistent (and in many cases, asymptomatic) infections these symbionts may be tolerated by, or act to dampen, *D. discoideum* pathogen clearance mechanisms (Brock et al., 2016). It may be that certain phage-symbiont pairings will result in more effective symbiont clearance. For example, in Pa1045 infected amoeba, despite detecting extracellular symbionts in treated amoeba cultures (Figure 6A), phage treatment reduced intracellular infections to almost indetectable levels and improved amoeba host fitness. Although we did not carry these amoeba lines forward in the serial transfer experiment, this symbiont might represent a more pathogen-relevant context for assessing the interplay between phage and amoeba defense responses in pathogen targeting.

Phage-symbiont dynamics within amoeba populations may also reflect a balancing act driven by both co-evolutionary pressures and physiological constraints. A preliminary test on a small number of Bonzo treated Pb433 isolates hinted at emergence of variable phage resistant phenotypes, suggesting that evolution of resistance and counterselection may underly co-persistence (preliminary data not shown). However, host-specific features may also contribute to outcome dynamics (beyond immune system contribution mentioned above), as highlighted by other natural symbiont systems (Koskella et al., 2011; Xu et al., 2016). One compelling hypothesis is that intracellular symbionts are protected from phage predation and that this protection varies by cell-type (vegetative amoeba vs. spores), intracellular location, and/or symbiont-specific intracellular invasion and replication characteristics. For example, Paranha-induced reduction of Pb433, but not Ph171, could be related to different intracellular profiles between these two symbionts (Figure 6). Certainly, phage penetration into cells and tissues is an important factor for phage stability and targeting efficacy, and may be highly variable (Bichet et al., 2021; Fajardo-Lubian and Venturini, 2023; Nguyen et al., 2017). Ultimately, the observed dynamics in our system are likely to be shaped by a combination of phage, symbiont, host, and environmental characteristics (Castledine and Buckling, 2024).

In summary, this study demonstrates the feasibility of maintaining and analyzing tripartite interactions within the *Paraburkholderia*-amoeba symbiosis system while underscoring the inherent variability of these relationships. Our in-amoeba phage-symbiont pairings point to intricate links between phage, symbiont, and host traits, highlighting the need to unravel the underlying mechanisms shaping these dynamics. Moving forward, insights gleaned from our phage screening efforts will guide the optimization of future phage isolation strategies, enabling a deeper understanding of *Paraburkholderia* phages (and their relationship with *Paraburkholderia* host species) and expanding our toolkit of available phage isolates. Integrating a broader collection of characterized phage isolates with existing *Paraburkholderia*-amoeba resources will better enable identification of the genotypic and phenotypic determinants of interaction outcomes. Moreover, the ability to explore the influence of environmental conditions and long-term associations on outcomes in this system reinforces its utility as a model for understanding potential interaction trajectories relevant for more complex biological contexts. Together, these findings lay the groundwork for future studies aimed at dissecting the ecological and evolutionary dynamics of multipartite symbiosis.

## Data Availability

The original contributions presented in the study are publicly available. This data can be found at: https://figshare.com/articles/dataset/DiSalvo_et_al_Phages_specific_to_Paraburkholderia_symbionts_of_soil_amoeba_Frontiers_in_Micro_Submission_Nov_2024/27922041?file=50850939.

## Footnotes

^1^ https://phaster.ca/

## Supplementary material

SUPPLEMENTARY FIGURE 1 Bonzo treatment of Pb433 infected amoeba reduces infection prevalence under a wide range of estimated phage to symbiont multiplicity of infection ratios. Pb433 infection prevalence (rfp-positive spores) from fruiting bodies developed after plating 10^5^ Pb433rfp infected amoeba spores with 10^5^, 10^6^, 10^7^, and 10^8^ total Bonzo pfu’s (reflecting 0.1 to 100 MOIs under the assumption that Pb433rfp cells outnumber spores in the sample by 10) or with phage buffer control (untreated).

SUPPLEMENTARY TABLE 1 Bacterial strains used for phage screening and host range analysis. For the main symbiont-relevant collection, information on isolation region and sample type is included. To generate the cladogram used to organize bacterial strains in the host range figure, the AtpD synthase GenBank accession number is indicated (associated with each isolate or, when necessary, a closely related representative). H. Arboretum and MLBS refer to the Houston Arboretum and Mountain Lake Biological Station, respectively.
