# Supplementary figures and images for: Tracking tripartite interaction dynamics: isolation, integration, and influence of bacteriophages in the Paraburkholderia-Dictyostelium discoideum symbiosis system
Source: Front Microbiol. 2025 May 2;16:1537073. doi: 10.3389/fmicb.2025.1537073 (PMC12081417; doi:10.3389/fmicb.2025.1537073)

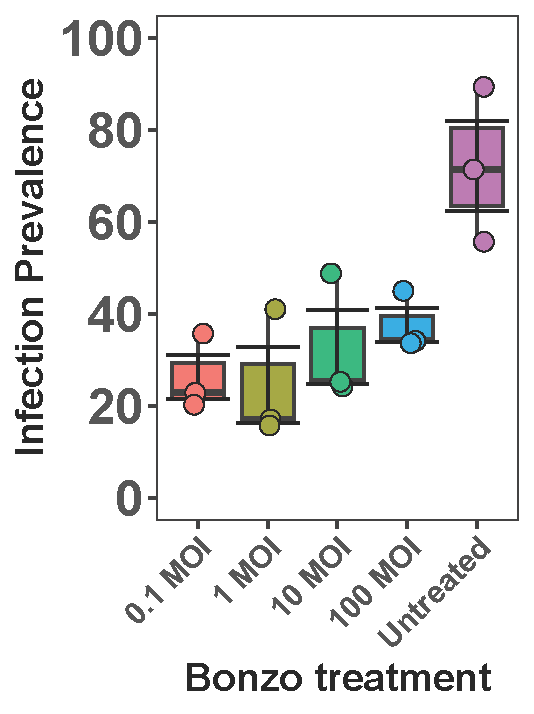

Supplement: SUPPLEMENTARY FIGURE 1 — Bonzo treatment of Pb433 infected amoeba reduces infection prevalence under a wide range of estimated phage to symbiont multiplicity of infection ratios. Pb433 infection prevalence (rfp-positive spores) from fruiting bodies developed after plating 105 Pb433rfp infected amoeba spores with 105, 106, 107, and 108 total Bonzo pfu’s (reflecting 0.1 to 100 MOIs under the assumption that Pb433rfp cells outnumber spores in the sample by 10) or with phage buffer control (untreated). [file Image_1.tiff]
