# Supplementary material for: Tracking tripartite interaction dynamics: isolation, integration, and influence of bacteriophages in the Paraburkholderia-Dictyostelium discoideum symbiosis system
Source: Front Microbiol. 2025 May 2;16:1537073. doi: 10.3389/fmicb.2025.1537073 (PMC12081417; doi:10.3389/fmicb.2025.1537073)
Supplement: SUPPLEMENTARY TABLE 1 — Bacterial strains used for phage screening and host range analysis. For the main symbiont-relevant collection, information on isolation region and sample type is included. To generate the cladogram used to organize bacterial strains in the host range figure, the AtpD synthase GenBank accession number is indicated (associated with each isolate or, when necessary, a closely related representative). H. Arboretum and MLBS refer to the Houston Arboretum and Mountain Lake Biological Station, respectively. [file Table_1.docx]

| **Genus** | **species** | **strain** | **Location** | **Host** | **GenBank** |
| --- | --- | --- | --- | --- | --- |
| *Paraburkholderia* | *agricolaris* | PaNC21 | North Carolina | *D. discoideum* | MH717907.1 |
| *Paraburkholderia* | *agricolaris* | Pa70 | Virginia-MLBS | *D. discoideum* | MH717908.1 |
| *Paraburkholderia* | *agricolaris* | Pa1060 | Virginia-MLBS | *D. discoideum* | MH717910.1 |
| *Paraburkholderia* | *agricolaris* | Pa1007 | Virginia-MLBS | *D. discoideum* | MH717911.1 |
| *Paraburkholderia* | *agricolaris* | Pa317 | Texas-H. Arboretum | *D. discoideum* | MH717912.1 |
| *Paraburkholderia* | *agricolaris* | Pa80 | Texas-H. Arboretum | *D. discoideum* | MH717914.1 |
| *Paraburkholderia* | *agricolaris* | Pa159 | Virginia-MLBS | *D. discoideum* | MH717917.1 |
| *Paraburkholderia* | *agricolaris* | Pa31 | Texas-H. Arboretum | *D. discoideum* | MH717921.1 |
| *Paraburkholderia* | *agricolaris* | Pa175 | Texas-H. Arboretum | *D. discoideum* | MH717933.1 |
| *Paraburkholderia* | *agricolaris* | Pa183 | Virginia-MLBS | *D. discoideum* | MH717934.1 |
| *Paraburkholderia* | *agricolaris* | Pa1045 | Virginia-MLBS | *D. discoideum* | MH717949.1 |
| *Paraburkholderia* | *bonniea* | Pb433 | Virginia-MLBS | *D. discoideum* | MH717913.1 |
| *Paraburkholderia* | *bonniea* | Pb395 | Virginia-MLBS | *D. discoideum* | MH717939.1 |
| *Paraburkholderia* | *bonniea* | Pb859 | Virginia-MLBS | *D. discoideum* | MH717946.1 |
| *Paraburkholderia* | *hayleyella* | Ph11 | Virginia-MLBS | *D. discoideum* | MH717909.1 |
| *Paraburkholderia* | *hayleyella* | Ph21 | Virginia-MLBS | *D. discoideum* | MH717918.1 |
| *Paraburkholderia* | *hayleyella* | Ph155 | Virginia-MLBS | *D. discoideum* | MH717930.1 |
| *Paraburkholderia* | *hayleyella* | Ph171 | Virginia-MLBS | *D. discoideum* | MH717932.1 |
| *Paraburkholderia* | *hayleyella* | Ph530 | Indiana-Bloomington | *D. discoideum* | MH717942.1 |
| *Paraburkholderia* | *hayleyella* | Phnc34 | North Carolina | *D. discoideum* | MH717952.1 |
| *Paraburkholderia* | *hayleyella* | Phnc63 | North Carolina | *D. discoideum* | MH717953.1 |
| *Burkholderia* | *xenovorans* | LB400 |  | free-living/soil | HQ398466.1 |
| *Burkholderia* | *fungorum* | Pf463 |  | Fungal | HQ398436.1 |
| *Burkholderia* | *tuberum* | Bt |  | Legume nitrofix | LR694404.1 |
| *Burkholderia* | *cepacia* | Bc25416 |  | Onion UCB717 | HQ398421.1 |
| *Burkholderia* | *silvatlantica* | Bspva5 |  | Plant beneficial | HQ398453.1 |
| *Burkholderia* | *unamae* | Bu |  | Plant beneficial | HQ398464.1 |
| *Burkholderia* | *insecticola* | RPE75 |  | Insect |  |
| *Caballeronia* | *beanbug* | SQ4a |  | Insect |  |
| *Rhizobium* | *sp.* | RhizSoil | Virginia-MLBS | free-living/soil |  |
| *Serratia* | *marcescens* | Ser454 |  |  |  |
| *Escherichia* | *coli* | Ecor64 |  |  |  |

Table 1
